# The Prognostic, Diagnostic, and Therapeutic Potential of TRAIL Signalling in Cardiovascular Diseases

**DOI:** 10.3390/ijms24076725

**Published:** 2023-04-04

**Authors:** Elaina Kelland, Manisha S. Patil, Sanjay Patel, Siân P. Cartland, Mary M. Kavurma

**Affiliations:** 1 Heart Research Institute, The University of Sydney, Sydney 2042, Australia; 2 Royal Prince Alfred Hospital, Sydney 2006, Australia

**Keywords:** TRAIL, diagnostic and therapeutic potential, atherosclerosis, heart failure, diabetes, clinical and pre-clinical studies

## Abstract

TNF-related apoptosis-inducing ligand (TRAIL) was originally discovered, almost 20 years ago, for its ability to kill cancer cells. More recent evidence has described pleiotropic functions, particularly in the cardiovascular system. There is potential for TRAIL concentrations in the circulation to act as prognostic and/or diagnostic factors for cardiovascular diseases (CVD). Pre-clinical studies also describe the therapeutic capacity for TRAIL signals, particularly in the context of atherosclerotic disease and diseases of the myocardium. Because diabetes mellitus significantly contributes to the progression and pathogenesis of CVDs, in this review we highlight recent evidence for the prognostic, diagnostic, and therapeutic potential of TRAIL signals in CVDs, and where relevant, the impact of diabetes mellitus. A greater understanding of how TRAIL signals regulate cardiovascular protection and pathology may offer new diagnostic and therapeutic avenues for patients suffering from CVDs.

## 1. Introduction

Cardiovascular disease (CVD) is an umbrella term for a group of disorders related to the heart and blood vessels and is the leading cause of death worldwide. Since 2019, >500 million cases of CVD were reported globally, with 18.6 million CVD-associated deaths [1]. Acute events caused by CVD include heart attack, stroke, and gangrene, which is a direct result of atherosclerosis, where blood vessels become blocked due to a fibro-fatty plaque that reduces nutrients and oxygen to the heart, brain, and lower limbs. Damage to the blood vessels can also promote heart failure, affecting the heart’s ability to pump blood around the body. Alarmingly, metabolic derangements such as diabetes mellitus and obesity significantly impact and contribute to the prevalence of CVDs. For example, the risk of ischaemic CVDs including myocardial infarction (MI) and stroke is increased by more than 50% with type-2 diabetes; diabetes and obesity increase the risk of heart failure by 112% and 65%, respectively [2,3]. Greater comprehension of CVD development, pathogenesis and early detection, and impact of metabolic diseases is critical for finding new strategies to reduce this burden.

A member of the TNF family of cytokines, TNF-related apoptosis-inducing ligand (TRAIL) was first discovered for its ability to kill human cancer cells upon ligation with its signalling receptors, namely, death receptor-4 and -5 (DR4 and DR5), without affecting normal cells [4,5]. Mice only have one death receptor, mDR5 (homologous to both human receptors), and similar to its actions in humans, TRAIL binding to mDR5 reduced tumours [6]. Because of these findings, TRAIL was hailed a potential cancer therapeutic; however, clinical trials in patients showed little survival benefit [7]. It is now clear that TRAIL signals have pleiotropic effects, like most TNF cytokines. In addition to apoptosis, TRAIL can stimulate necroptosis [8] and autophagy [9], as well as cell survival processes such as proliferation, migration, and differentiation [10,11,12,13,14]. Human decoy receptors (DcR) for TRAIL have also been identified and include DcR1, DcR2, and the soluble receptor osteoprotegrin (OPG). Upon binding TRAIL, they inhibit the induction of apoptosis [15], yet how these receptors impact non-apoptotic function(s) of TRAIL is not clear.

TRAIL signalling in CVD has gained considerable interest, and the evidence in atherosclerotic disease mostly points towards a protective role, particularly since reduced circulating TRAIL levels are associated with increased cardiovascular events and mortality [16,17]. TRAIL’s role in the heart is conflicting. In some circumstances it may protect, while in others it may contribute to pathogenesis. Furthermore, it is unclear as to whether TRAIL is a risk factor or a risk marker in CVDs [18]. This review summarises our current understanding of TRAIL signals in atherosclerotic vascular disease as well as conditions of the heart. A better understanding of how TRAIL signalling regulates cardiovascular protection and pathology may offer new diagnostic and therapeutic avenues for patients suffering from CVDs.

## 2. Atherosclerosis and Vessel Diseases

**Figure 1:**
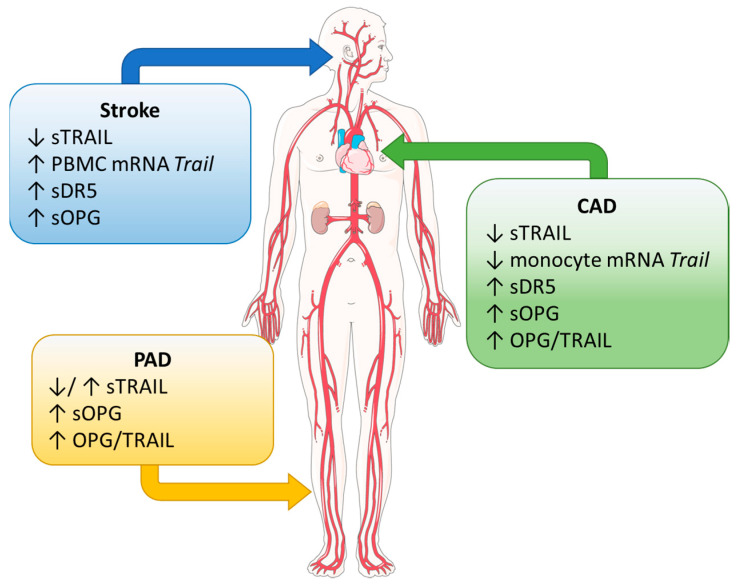
Summary of circulating TRAIL and TRAIL receptor levels in atherosclerotic vascular diseases including coronary artery disease (CAD), stroke, and peripheral artery disease (PAD); s, soluble; ↑, increased; ↓, decreased. Details are within the text.

**Figure 2:**
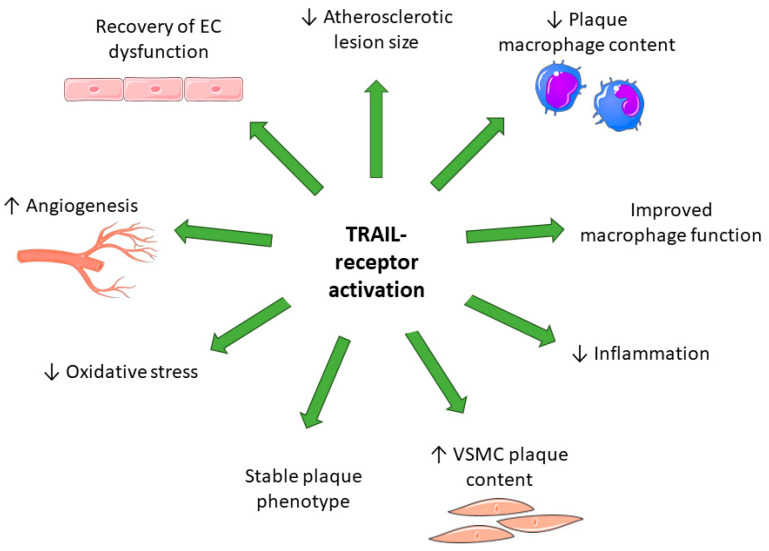
Therapeutic potential of TRAIL signals in atherosclerotic disease. Activating TRAIL signalling receptors in the vessel wall improve multiple features of atherosclerosis and diabetic-atherosclerotic disease. EC, endothelial cell; VSMC, vascular smooth muscle cells; ↑, increased; ↓, decreased. Details are within the text.

Atherosclerosis is the pathological process underlying coronary heart disease (e.g., coronary artery disease (CAD)), stroke, and peripheral artery disease (PAD). It is a condition where vascular smooth muscle cells (VSMCs), inflammatory cells (e.g., monocytes, macrophages), lipids, cholesterol, and cellular waste accumulate, producing a thickened plaque in the arterial wall. Because the endothelium has anti-inflammatory, anti-thrombotic, and anti-atherosclerotic properties, one of the earliest indications for atherogenesis is endothelial dysfunction, with diabetes mellitus precipitating this process [19]. As the lesion grows, the plaque can become vulnerable and rupture. Plaque rupture is the major complication and cause of CVD mortality and morbidity. Below we have summarised our current knowledge of TRAIL biology in the context of atherosclerotic disease, specifically focusing on findings implicating a prognostic/diagnostic and therapeutic role (Table 1 and Table 2; Figure 1 and Figure 2). Where relevant, the impact of diabetes mellitus is also highlighted.

### 2.1. Diagnostic and Prognostic Potential

(i) CAD—The occlusion of blood vessels to the heart by atherosclerotic lesions leads to reduced blood flow to the myocardium, leading to MI and potentially heart failure. Several reports showed that circulating TRAIL levels were lower in patients diagnosed with CAD vs. those undiagnosed [20,21,22,23], associating with oxidative stress [67] and disease severity [16,17]. Interestingly, monocyte *Trail* mRNA was reduced in CAD, concomitant with circulating levels from the same patients, implicating monocytes as a significant source of TRAIL in healthy circulation and compromised in CAD [23]. The Canakinumab Anti-inflammatory Thrombosis Outcomes Study identified IL-18 and IL-6 as associated with residual inflammatory risk following IL-1β inhibition in patients [89]. A negative association between IL-18 and TRAIL, but not IL-6, was identified with CAD [23], and colchicine anti-inflammatory treatment increased plasma TRAIL levels in these patients [61], supporting TRAIL’s anti-inflammatory role. Because diabetes mellitus is a common comorbidity of atherosclerosis, lower levels of circulating TRAIL were also reported in type-2 diabetes [90,91]; however, TRAIL levels did not reflect early stages of atherosclerosis (as a measure of carotid artery intima-media thickness) in these patients [92].

The prognostic potential of TRAIL has been reported. In the InCHIANTI study, a population-based study of aging, almost 1300 individuals >65 years of age were selected randomly, and blood was sampled at baseline and at 3 and 6 years later. A strong and independent association between low TRAIL levels and all-cause mortality over a period of 6 years was found in participants with pre-existing CVDs (including and not limited to heart failure, MI, stroke, and PAD) [16]. In fact, the authors reported that participants with TRAIL levels <84.5 pg/mL at baseline had 2–3-fold greater risk of cardiovascular mortality [16]. A similar finding was observed in a second smaller prospective study; TRAIL serum levels were a strong predicter of death [17]. These findings suggest that measuring TRAIL levels in patients with CVD has prognostic value and could be considered as a measure of cardiovascular risk.

TRAIL receptors have also been linked to CAD. For example, a recent biomarker study retrospectively examined two clinical trials and identified DR5 as one of 18 proteins increased with CAD [24], suggesting that it may act as a potential diagnostic marker. DR5 may also have prognostic value since higher levels are associated with all-cause mortality in acute MI patients, along with growth development factor-15, and in combination with established risk factors predicted survival with 88% accuracy [25]. Increased DR5 levels may also have prognostic potential in chronic kidney disease. Indeed, DR5 was identified as a predictor of cardiovascular mortality, as well as a predictor of MI and heart failure readmission [26,27], a finding also observed in diabetes [28].

Several studies suggest that increased concentrations of OPG are associated with increased cardiovascular risk. For example, the Tromsø Study, a large population-based cohort study of 6265 participants, found high levels associated with increased risk of MI, stroke, total mortality and mortality of ischaemic heart disease, stroke, and non-vascular conditions [29]. These were independent of cardiovascular risk factors [29]. OPG levels may be independently associated with traditional cardiovascular risk factors, including diabetes [30,31]. High OPG levels were also reflective of disease severity and predicted cardiovascular events and all-cause mortality in CAD patients [30,33]; however, how this impacts TRAIL signalling is unclear. The OPG/TRAIL ratio may have prognostic potential; higher OPG/TRAIL ratios were observed in CAD [21] and predicted all-cause cardiovascular mortality in patients with renal failure [32]. In contrast, a single OPG measurement was deemed insufficient to diagnose CAD in patients with angina [33].

(ii) Stroke—Similar to coronary heart disease, studies report lower levels of circulating TRAIL in patients that had a stroke vs. healthy individuals [34,35,36,37,38,39], and in some cases (but not all) it was associated with stroke severity [34,35,37,39]. The two stroke subtypes involving active atherosclerosis are large artery atherosclerosis (LAA), often in the carotid artery, and small artery occlusion (SAO). Conflicting evidence exists as to whether TRAIL levels differ significantly between subtypes, some reporting no change [34,36]; however, one study identified that circulating TRAIL levels were lower in patients with SAO [38]; low levels persisted for at least three months after the onset of stroke. Peripheral blood mononuclear cells (PBMCs) were also assessed for TRAIL expression from patients with ischaemic stroke, and while serum TRAIL levels were reduced, this did not reflect PBMC mRNA expression, which was increased at the time of admission [36]. The impact of PBMC-derived TRAIL vs. serum TRAIL in stroke is still unclear. DR5 and OPG levels may also be relevant. For example, DR5 is increased in the circulation of symptomatic patients with carotid plaque, associated with increased DR5 plaque protein when compared to asymptomatic patients [40], suggesting that levels may reflect the severity of disease. DR5 and OPG levels were also associated with stroke, and both were elevated in LAA patients compared to controls [35]. Furthermore, increased OPG levels (assessed at the time of admission) predicted poorer prognosis and mortality of patients who suffered an ischaemic stroke [41,42].

(iii) PAD—There is limited and conflicting data as to whether TRAIL could act as a prognostic marker factor for PAD. Serum TRAIL levels were reduced in type-2 diabetic nephropathy patients with foot ulcers vs. those without [43], a finding also observed in diabetic PAD patients alone [44]. In contrast, O’Sullivan et al. reported higher levels of TRAIL in PAD [45]. DR4 and DR5 measurements from PAD patients have not been described; however, increased levels of circulating OPG were evident in PAD and type-2 diabetic PAD patients [44,45]. Further, the OPG/TRAIL ratio was described to be higher in both cohorts [44]. More work is needed to understand the contribution of TRAIL and its receptors to PAD.

### 2.2. Therapeutic Potential for Atherosclerotic Disease—Teachings from In Vitro and Pre-Clinical Studies

In vitro cell studies and pre-clinical animal models have shown that TRAIL plays important role(s) in the development of atherosclerosis, either protecting or contributing to pathogenesis (Figure 2). In response to peri-vascular cuff injury, *Trail*^−/−^ mice had reduced neointimal hyperplasia compared to *Trail*^+/+^ mice, and recombinant TRAIL delivery recovered neointimal thickening [10], a finding supported by in vitro studies using human VSMCs [10,14,81]. These findings suggest that TRAIL may contribute to the development of early atherosclerosis. Because TRAIL promotes VSMC migration into the plaque, this process may also contribute to plaque stability in advanced lesions, reducing the incidence of rupture [70]. Indeed, atherosclerotic *Trail*^−/−^*Apoe*^−/−^ mice developed a larger [23,65,69], more macrophage-rich plaque of unstable phenotype with reduced VSMC and collagen content [23,65,66,69]. Mice lacking TRAIL had greater vascular oxidative stress [67], inflammation [23,65,68], and endothelial dysfunction [67] compared to the control. Importantly, metabolic derangements were observed; *Trail*^−/−^*Apoe*^−/−^ mice developed features of type-2 diabetes [65,68]. *Trail*^−/−^ mice or neutralisation of TRAIL in mice resulted in increased susceptibility to streptozotocin-induced diabetes or high fat diet-induced insulin resistance [63,64]. TRAIL’s expression in the vessel wall is controlled by insulin. We showed that chronic exposure of human VSMCs to insulin suppressed TRAIL gene expression, promoting apoptosis [80]. TRAIL expression was also downregulated in vessels of diabetic rats [93]. Furthermore, microarray analysis identified *Glut1,* a glucose transporter, as a pathological gene upregulated in aortic tissues of *Trail*^−/−^ mice [61]. These pre-clinical findings support TRAIL’s involvement in metabolic CVDs and provide insight into the impact of TRAIL suppression in people. Given that TRAIL can regulate the vascular system in diabetes [94], a greater understanding of TRAIL signalling in the progression of diabetic CVD is needed.

To understand why TRAIL was suppressed in CAD, we found that elevated levels of IL-18 repressed TRAIL transcription and gene expression in healthy human monocytes by inhibiting NFκB’s ability to bind the TRAIL promoter [23]. Indeed, macrophages lacking TRAIL were more inflammatory, less effective in their ability to efferocytose, showed impaired cholesterol handling, and had reduced migratory ability [23], which are hallmarks of dysfunctional macrophages in lesions, accelerating atherosclerosis [23,95]. In contrast, exogenous TRAIL pre-treatment increased lipid uptake and foam cell formation and contributed to macrophage apoptosis [82]. The exogenous delivery of TRAIL in pre-clinical models of atherosclerosis has been described and for the most part shows promising therapeutic potential. Administration of TRAIL protein, TRAIL gene therapy, or TRAIL bone marrow transplantation attenuated atherosclerosis development, reduced macrophage content in the vessel wall, and reduced inflammation in diabetic *Apoe*^−/−^ or *Trail*^−/−^*Apoe*^−/−^ mice [23,70].

As described earlier, the vascular endothelium is critical for the maintenance of cardiovascular homeostasis. Although reports indicate that TRAIL can stimulate apoptosis of endothelial cells (ECs) [86], the majority of findings reports on increased EC survival processes, particularly at physiological concentrations. For example, diabetes-induced endothelial dysfunction was improved by TRAIL, in part via its ability to increase endothelial nitric oxide synthase (eNOS) production [73]. TRAIL also prevented high glucose-induced apoptosis of ECs and protected against angiotensin II (AngII)- or TNF-α-induced oxidative stress, which are pro-atherogenic conditions [67,73,83]. Furthermore, TRAIL reduced AngII-induced endothelial reactivity and monocyte adhesion, improving endothelial integrity [67]. These findings further support TRAIL’s therapeutic potential in the vasculature.

In addition to their multiple functions in maintaining vascular homeostasis is the regenerative capacity of ECs and their ability to develop new blood vessels by angiogenesis, an essential process that is upregulated during ischemia to increase blood perfusion. However, in CVDs, endogenous angiogenic processes are impaired, contributing to vascular insufficiency. In vitro, TRAIL stimulated EC proliferation, migration, and differentiation [13,62,84], processes important for the formation of vascular tubules. We used the hindlimb ischemia model of PAD; *Trail*^−/−^ mice had impaired limb movement and increased limb necrosis associated with markedly reduced (~70%) capillary density in limb tissues [62]. Viral TRAIL gene therapy dramatically improved limb blood perfusion and vascularisation mediated by NOX4-inducible eNOS phosphorylation and generation of nitric oxide [62], a key factor regulating vessel patency. This effect may be a consequence of TRAIL interacting with its receptor mDR5 [62], although the exact contribution of TRAIL receptor(s) to ischemia-induced angiogenesis is unknown. In contrast, TRAIL stimulated apoptosis in the human brain endothelial cell line, hCMEC/d3 [85], suggesting organ- and cell-specific effects. Indeed, amyloid-beta (Aβ), associated with neurodegeneration and known to accumulate after stroke or in cerebral ischemia [96,97], interacted with DR4 and DR5, triggering the activation of caspase-8 and mitochondrial pathways for apoptosis in these cells [98].

The impact of TRAIL receptors in atherosclerosis in pre-clinical studies is less clear. Brachiocephalic arteries of *Apoe*^−/−^ mice express OPG [71]; its expression was associated with lesions that are unstable [99]. However, *Opg*^−/−^*Apoe*^−/−^ mice had increased lesion area, with a 40% reduction in plaque cellularity compared to *Apoe*^−/−^ mice [72]. The exogenous treatment of VSMCs promoted survival in vitro, supporting this finding. Furthermore, the increased lesion size was a result of calcification and extracellular matrix deposition [72]. The authors suggested that reduced MMP-9 activity could contribute to increased matrix deposition in *Opg*^−/−^*Apoe*^−/−^ plaque [72]. These findings imply that OPG has a complex role in atherogenesis, and more work is needed to understand its contribution and whether it can be targeted for its therapeutic potential.

## 3. TRAIL Signalling in the Myocardium

**Figure 3:**
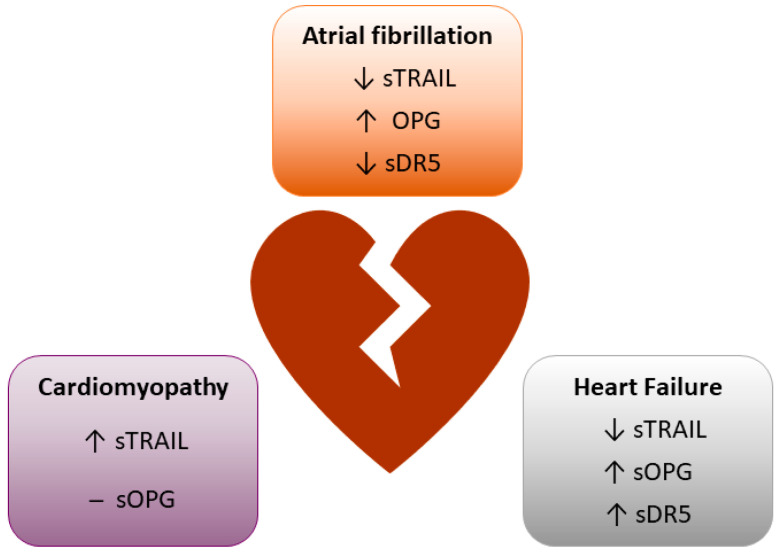
Summary of soluble (s) concentrations and expression of TRAIL and TRAIL receptors in diseased myocardium; s, soluble; ↑, increased; ↓, decreased. Details are within the text.

**Figure 4:**
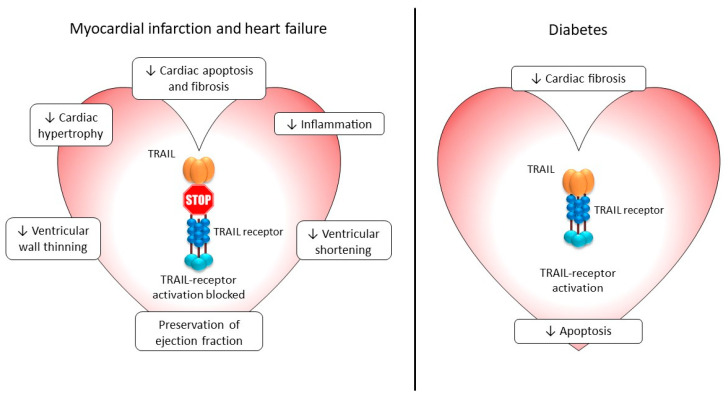
Therapeutic potential of TRAIL signals in the myocardium. Blocking TRAIL’s interaction with its signalling receptor(s) in pre-clinical models of heart failure and MI improve multiple functions of the myocardium. In contrast, activating TRAIL signals in the diabetic heart protects against cardiac fibrosis and apoptosis; ↑, increased; ↓, decreased. Details within the text.

Injury to the myocardium due to multiple factors, e.g., ischemia, atherosclerosis, infections, etc., can result in heart failure, a progressive disease that impacts the heart’s ability to adequately pump blood around the body, manifesting impaired cardiac function, disturbed electrical activity, and abnormal tissue architecture. Diabetes mellitus increases the risk of heart failure and may also contribute to the progression of cardiomyopathy and atrial fibrillation [100,101], two related pathologies. Below is a summary of the current findings examining the diagnostic/biomarker potential of TRAIL signals in the myocardium (Table 1 and Figure 3). We also describe in vitro and pre-clinical findings that show the therapeutic potential of TRAIL signals (Table 2 and Figure 4), and where possible, we describe these in the context of metabolic disease.

### 3.1. Diagnostic and Prognostic Potential

(i) Heart Failure—Some studies have linked circulating levels of TRAIL or its receptors to heart failure. For example, a strong inverse association of all-cause mortality was observed in advanced heart failure with TRAIL, whereas higher levels of TRAIL reflected better prognoses [46]. In support, another study identified TRAIL as one of five multi-biomarkers that could predict patient mortality [47]. Furthermore, a negative association between TRAIL levels and all-cause mortality and hospitalisation was identified in patients with preserved ejection fraction, whereas a positive association with circulating DR5 was found [48]. In contrast, a prospective observational study showed no difference in TRAIL levels in heart failure patients undergoing cardiac resynchronisation therapy, and TRAIL levels did not predict mortality [49]. Circulating DR5 is increased in heart failure patients with worse left ventricular ejection fraction and diastolic function but positively associating with the incidence of disease [48,50]. The decoy receptor OPG has also been linked to chronic heart failure with increased levels observed in patients [51], associated with adverse outcomes within 2 years [52]. OPG levels were also shown to be a significant predictor of mortality [53]. These findings suggest that TRAIL and TRAIL receptors may act as a potential biomarker in heart failure as well as predict patient outcomes and mortality; however, more studies are needed to confirm these.

(ii) Cardiomyopathies—Non-ischaemic dilated cardiomyopathy is a condition that causes hypertrophy of the ventricles, effecting myocardial contractility and reducing the ejection fraction, leading to heart failure if left untreated. Plasma TRAIL levels were upregulated in patients with non-ischaemic dilated cardiomyopathy and positively correlated with left ventricular end-diastolic diameter, whereas OPG levels remained unchanged [54]. TRAIL levels were also altered in patients with Chagas cardiomyopathy, an inflammatory disease caused by the protozoan *Trypanosoma cruzi*, which can progress to dilated cardiomyopathy and heart failure. Patients suffering from severe Chagus had elevated levels of circulating TRAIL, correlated with left ventricular ejection fraction and left ventricular diastolic diameter [55]. Whether TRAIL and its receptors act as diagnostic or prognostic factors in cardiomyopathy is unclear.

(iii) Atrial fibrillation (AF) is a common atrial arrhythmia that can be paroxysmal (~1 week), persistent (>1 week), or permanent and can increase the risk of stroke and heart failure. A prospective observational study identified circulating TRAIL levels to be decreased in patients with successful ablation of AF [56]. Conversely, low levels of circulating TRAIL were evident in acute onset AF, and they were increased following sinus rhythm maintenance [58]. Another study found no differences in plasma TRAIL levels observed in patients with or without AF recurrence; however, when the transcardiac gradient was measured, TRAIL levels were reduced, revealing this gradient to be a negative predictor for AF recurrence [57]. Like TRAIL, DR5 levels are reduced in AF; however, no links have been described between AF and sinus rhythm [59]. As for decoy receptors, very little information exists. There is some evidence linking OPG to AF; OPG expression was increased in samples of the right atrial appendage from persistent and paroxysmal AF patients vs. normal controls and sinus rhythm patients [60]. Whether TRAIL and its receptors can act as prognostic factors or biomarkers requires further elucidation.

### 3.2. Therapeutic Potential for Diseases of the Heart—Teachings from In Vitro and Pre-Clinical Studies

TRAIL and its receptors are expressed in normal and diseased human and rodent hearts at varying levels [51,74,102,103], although the impact of TRAIL signals in the heart is not fully elucidated. Apoptosis and proliferation play key roles under normal and pathogenic conditions, but it is not clear if TRAIL play a protective or detrimental role here. Administration of TRAIL or a small molecule DR5 agonist (bioymifi) to cardiomyocytes in vitro did not induce apoptosis or affect cell viability, but it altered cardiomyocyte structure, promoting hypertrophy in an ERK1/2-dependent manner [75]. Similarly, the administration of recombinant TRAIL or adenoviral TRAIL resulted in a significant reduction in cardiac fibrosis and apoptosis compared to control diabetic animals [76], and MD5-1 (agonistic mDR5 mAb) treatment to wildtype mice resulted in increased heart weight and cardiomyocyte area, in part through the activation of the epidermal growth factor receptor [75]. Increased ventricular fractional shortening was also observed with DR5 activation [75]. OPG may also associate with cardiomyocyte hypertrophy, since hearts from *Apoe*^−/−^ mice had increased cardiomyocyte diameter associated with increased OPG protein [77]. Further, OPG delivery to spontaneously hypertensive rats resulted in enlarged cardiomyocytes and fibroblasts, with OPG regulating cardiac and fibrosis-related proteins [78]. These findings suggest that the activation of TRAIL signals via its signalling receptors may regulate structural changes in the heart under physiological conditions and in conditions of diabetic cardiomyopathy. Whether the actions of OPG are TRAIL-dependent or -independent is unclear.

Other studies report opposing actions of TRAIL in the heart. For example, TRAIL stimulated, whereas neutralising DR5 inhibited, the stretch-induced apoptosis of cardiomyocytes [87]. The increased expression of DR4/DR5 was also observed in doxorubicin-treated human cardiomyocytes, associated with spontaneous apoptosis [88]. Apoptotic cell death is increased in heart failure and may contribute to unfavourable left ventricular remodelling [104]. Blocking DR5 using a soluble immunoglobulin fusion protein (sDR5-fc) in a heart failure model that prevents cardiac cell death and inflammation, preserves ejection fraction and fractional shortening, reduces fibrosis, and prevents ventricular wall thinning, findings observed in rodents, pigs, and monkeys [79]. Silencing DR5 in an MI model in rats also reduced myocardial damage and infarct size, and it reduced the cardiac expression of apoptotic mediators [74]. These imply that under certain conditions, the activation of TRAIL signals in the heart could be detrimental, and a blockade of TRAIL signalling may be used as a potential therapeutic. More research is needed to fully comprehend the diverse roles of TRAIL and its receptors in cardiac function under normal and pathological conditions.

## 4. Conclusions

Targeting the TRAIL pathway in CVDs holds great prognostic and diagnostic potential. TRAIL concentrations are suppressed, whereas TRAIL receptor levels are increased in people with CVDs, which are associated with cardiovascular risk. Pre-clinical models have also identified that TRAIL signals also play a role in disease protection or progression, offering new therapeutic possibilities for the treatment of CVDs. Promoting TRAIL-receptor activation in atherosclerotic disease could be beneficial; drugs already in use to activate TRAIL signals in clinical trials in cancer could be repurposed or modified for atherosclerosis. On the other hand, novel therapeutics aimed at blocking TRAIL signalling in the myocardium could improve heart failure. More research is needed to fully comprehend the role of TRAIL and its receptors in atherosclerotic vessel diseases and the myocardium.

## Figures and Tables

**Table 1:** Summary of clinical findings and evidence of TRAIL signalling in CVDs.

Disease	Protein	Finding	Reference
CAD	TRAIL	Decreased circulating TRAIL in patients with CAD	[20,21,22,23]
		Negative association of circulating TRAIL with disease severity	[16,17]
		Reduced expression of TRAIL on monocytes from CAD patients	[23]
	DR5	Increased circulating DR5 identified as a potential prognostic factor in all-cause mortality in MI patients, cardiovascular mortality, and MI and heart failure readmission in chronic kidney disease and diabetes patients	[24,25,26,27,28]
	OPG	Positive association of circulating OPG with risk of all-cause and cardiovascular event-associated mortality	[29,30]
		Increased OPG associated with cardiovascular risk factors, e.g., diabetes	[30,31]
		Increased OPG a predictor for all-cause mortality in patients with renal failure	[32]
		Single OPG measurement insufficient to diagnose CAD in patients with angina	[33]
Stroke	TRAIL	Decreased circulating TRAIL in stroke patients	[34,35,36,37,38,39]
		Circulating levels associated with stroke severity	[34,35,37,39]
		Increased expression of TRAIL on monocytes with reduced circulating TRAIL at stroke onset	[36]
	DR5	Increased DR5 in carotid plaques and circulation of symptomatic patients	[40]
		Elevated in LAA stroke	[35]
	OPG	Elevated in LAA stroke	[35]
		High levels at time of admission predicts poorer prognosis and mortality in ischemic stroke	[41,42]
PAD	TRAIL	Circulating TRAIL reduced in patient with diabetic complication, i.e., foot ulcers and PAD	[43,44]
		Increased circulating TRAIL in patients with PAD and diabetes compared to diabetes alone	[45]
	OPG	Increased in PAD and diabetic PAD; associated with decreased TRAIL	[44]
		Increased in PAD and diabetic PAD; associated with higher TRAIL	[45]
Heart failure	TRAIL	Inverse association of circulating TRAIL and all-cause mortality and hospitalisation	[46,47,48]
		TRAIL did not predict mortality in heart failure patients undergoing cardiac resynchronisation therapy	[49]
	DR5	Positive correlation between plasma DR5 and HF incidence, preserved ejection fraction and left ventricular ejection fraction	[48,50]
	OPG	Positive association with circulating OPG and prediction of adverse outcomes and mortality	[51,52,53]
Cardiomyopathy	TRAIL	Increased systemic TRAIL in dilated cardiomyopathy patients	[54]
		Positive association with circulating TRAIL and left ventricular ejection fraction and left ventricular diastolic diameter	[55]
	OPG	Increased in the myocardium of dilated cardiomyopathy patients, but no systemic change	[54]
Atrial fibrillation	TRAIL	Reduced circulating TRAIL with successful ablation of AF	[56]
		Reduced trans cardiac gradient of TRAIL with AF recurrence	[57]
		Not useful in predicting the return to sinus rhythm	[58]
	DR5	Inverse association of DR5 with AF, but no difference in concentration between patients in sinus rhythm and in AF	[59]
	OPG	Identified an increasing gradient of atrial expression of OPG with increasing degrees of AF	[60]
		Not useful in predicting the return to sinus rhythm	[58]

Footnote: CAD, coronary artery disease; MI, myocardial infarction; LAA, large artery atherosclerosis; PAD, peripheral artery disease; HF, heart failure; AF, atrial fibrillation.

**Table 2:** Pre-clinical findings implicating TRAIL signals in cardiovascular disease.

Model	Animal/Cell Type	Model/Treatment	Finding	Reference
In vivo	*Trail*^−/−^ mice	Chow	Upregulation of glucose transporter *Glut1* in aortic tissue by microarray	[61]
		Peri-vascular cuff; intimal thickening	Reduced intimal thickening; recombinant TRAIL recovered the neointima after cuff placement in *Trail*^−/−^ mice; TRAIL stimulates VSMC proliferation and migration in vivo	[10]
		HLI	Reduced vascularisation after HLI; TRAIL gene therapy; improved limb perfusion and vascularisation	[62]
		Western diet	Insulin resistance; increased vascular inflammation	[63]
		STZ-induced diabetes	Increased susceptibility to STZ-induced diabetes	[64]
	NOD mice	CY-induced diabetes	Neutralising TRAIL by soluble TRAIL-R enhanced CY-induced diabetes	[64]
	*Trail*^−/−^*Apoe*^−/−^ mice	Atherosclerosis; Western diet	Developed larger, macrophage-rich plaques of unstable phenotype (thin cap, large necrotic core, reduced VSMC and collagen content); developed features of type-2 diabetes	[65]
		Atherosclerosis; cholate free Western diet	Developed larger atherosclerotic plaque	[66]
		Atherosclerosis; Western diet; bone marrow transplant	TRAIL-expressing bone marrow attenuated atherosclerosis; reduced inflammation	[23]
		Atherosclerosis; Western diet	Increased vascular oxidative stress; increased aortic endothelial dysfunction	[67]
		Atherosclerosis; Western diet	Increased inflammation; diabetic nephropathy	[68]
		Atherosclerosis; Western diet	Increased plaque; calcification	[69]
	*Apoe*^−/−^ mice	STZ-induced diabetes	Attenuation of atherosclerotic plaque with recombinant TRAIL or adenoviral TRAIL; reduced plaque macrophage content	[70]
		Chow	OPG expressed in the brachiocephalic arteries, associated with chondrocyte-like cells	[71]
	*Opg*^−/−^*Apoe*^−/−^ mice	Atherosclerosis; chow	Increased atherosclerosis and calcification; reduced plaque cellularity	[72]
	Rats	STZ-induced diabetes	Endothelial dysfunction was attenuated with recombinant TRAIL treatment	[73]
		Acute myocardial infarction	Soluble DR5 reduced infarct size, myocardial damage, and expression of apoptotic mediators	[74]
	C57BL/6 mice	MD5-1 antibody and Bioymifi (small molecule DR5 agonist)	DR5 activation increased heart weight, cardiac hypertrophy, left ventricular ejection fraction, and fractional shortening	[75]
	*Apoe*^−/−^ mice	STZ-induced diabetes;	Recombinant TRAIL and AAV TRAIL reduced cardiac fibrosis and apoptosis in diabetes	[76]
		STZ-induced diabetes	Increased OPG expression associated with cardiomyocyte hypertrophy	[77]
	Spontaneously hypertensive rats	Recombinant OPG	Increased left ventricular weight	[78]
	Rats, pigs and monkeys	Myocardial infarction	DR5 inhibition reduced infarct size, cardiomyocyte death, and fibrosis and prevented ventricular wall thinning; preserved ejection fraction and fractional shortening	[79]
In vitro	VSMC	Recombinant TRAIL	Increases proliferation and migration in human aortic VSMCs	[10]
		Recombinant TRAIL	Increases proliferation and migration via activation of insulin-like growth factor in human aortic VSMCs	[14]
		Insulin	Chronic insulin suppresses TRAIL expression and promotes apoptosis in human aortic VSMCs	[80]
		Recombinant PDGFB	Increases proliferation and migration via induction of TRAIL transcription and gene expression in human aortic VSMCs	[81]
	Monocyte/ macrophage	Recombinant IL-18	Suppressed TRAIL gene expression and transcription via blocking NFκβ binding to the TRAIL promoter in human monocytes	[23]
		Recombinant TRAIL	Increased lipid uptake and foam cell formation; macrophage apoptosis in RAW264.7 and THP-1 cells	[82]
		Basal, LPS and acLDL	*Trail*^−/−^ bone marrow-derived macrophages were more inflammatory and had a reduced ability to efferocytose; had impaired cholesterol and impaired ability to migrate compared to *Trail*^+/+^ bone marrow-derived macrophages	[23]
	ECs	Recombinant TRAIL	TRAIL treatment inhibited TNFα/hyperglycaemia-induced inflammation and ROS production in HAECs	[83]
			TRAIL inhibited high glucose-induced ROS and cell death, in part via Akt and eNOS phosphorylation in HUVEC	[73]
			TRAIL protected against AngII-induced oxidative stress; reduced AngII-induced monocyte adhesion and improved EC integrity by redistributing VE-cadherin expression to the cell surface	[67]
			Increased HMEC-1 proliferation, migration, and tubule formation via NOX-4-inducible eNOS phosphorylation and nitric oxide production	[62]
			Increased HMEC-1 proliferation, migration, and tubule formation	[13]
			Increased HUVEC migration, invasion, and tubule formation	[84]
			Increased apoptosis of HMEC/d3 cells	[85]
			Increased HUVEC apoptosis	[86]
	Cardiomyocytes	Recombinant TRAIL and Bioymifi	DR5 activation via EGFR increased ERK1/2 phosphorylation for hypertrophy; cell death or viability not affected	[75]
		AAV-OPG vector and OPG siRNA	OPG increased cell surface size and expression of hypertrophy proteins in rat cardiomyocytes	[78]
		Recombinant TRAIL and soluble DR5 (sDR5)	TRAIL increased, while sDR5 neutralised stretch-induced apoptosis in rat cardiomyocytes	[87]
		Doxorubicin	Increased DR4 and DR5 mRNA/protein associating with enhanced TRAIL-induced apoptosis in human induced pluripotent stem cell-derived cardiomyocytes	[88]

Footnote: AAV, adeno-associated virus; acLDL, acetylated low density lipoprotein; AngII, angiotensin II; CY, cyclophosphamide; EGFR, epidermal growth factor receptor; eNOS, endothelial nitric oxide synthase; ERK1/2, extracellular signal-regulated kinase 1/2; HAEC, human aortic endothelial cell; HLI, hindlimb ischemia; HMEC-1, human microvascular endothelial cell-1; HUVEC, human umbilical vein endothelial cell; IL-18, interleukin-18; LPS, lipopolysaccharide; NFκβ, nuclear factor κβ; NOX-4, NADPH oxidase-4; PDGFB, platelet-derived growth factor-B; ROS, reactive oxygen species; STZ, streptozotocin; VSMC, vascular smooth muscle cells.

## Data Availability

Data sharing not applicable.
